# Assisted reproductive technology in Japan: A summary report for 2020 by the ethics Committee of the Japan Society of obstetrics and gynecology

**DOI:** 10.1002/rmb2.12494

**Published:** 2023-01-04

**Authors:** Yukiko Katagiri, Seung Chik Jwa, Akira Kuwahara, Takeshi Iwasa, Masanori Ono, Keiichi Kato, Hiroshi Kishi, Yoshimitsu Kuwabara, Miyuki Harada, Toshio Hamatani, Yutaka Osuga

**Affiliations:** ^1^ Department of Obstetrics and Gynecology, Faculty of Medicine Toho University Tokyo Japan; ^2^ Department of Obstetrics and Gynecology Saitama Medical University Saitama Japan; ^3^ Department of Obstetrics and Gynecology, Graduate School of Biomedical Sciences Tokushima University Tokushima Japan; ^4^ Department of Obstetrics and Gynecology Tokyo Medical University Tokyo Japan; ^5^ Kato Ladies Clinic Tokyo Japan; ^6^ Department of Obstetrics and Gynecology The Jikei University School of Medicine Tokyo Japan; ^7^ Department of Obstetrics and Gynecology Nippon Medical School Tokyo Japan; ^8^ Department of Obstetrics and Gynecology, Graduate School of Medicine The University of Tokyo Tokyo Japan; ^9^ Department of Obstetrics and Gynecology School of Medicine, Keio University Tokyo Japan

**Keywords:** assisted reproductive technologies, fertility rate, in vitro fertilization, intracytoplasmic sperm injections, Japan

## Abstract

**Purpose:**

Since 1986, the Japan Society of Obstetrics and Gynecology assisted reproductive technology (ART) registry system has collected data on national ART use and outcomes trends in Japan. Herein, we describe the characteristics and outcomes of ART cycles registered during 2020 and compare the results with those from 2019.

**Methods and Results:**

In 2020, 621 ART facilities participated in the registration. The total number of registered cycles was 449 900, and there were 60 381 live births, which decreased from the previous year (1.79% and 0.36% decrease, respectively). The number of freeze‐all in vitro fertilization (IVF) and intracytoplasmic sperm injection (ICSI) cycles increased in 2020, and the number of neonates born was 2282 for IVF‐embryo transfer (ET) cycles and 2596 for ICSI cycles, which had decreased from the previous year. Frozen–thawed ET (FET) cycles had slightly increased from 2019 (0.04%). In 2020, 215 285 FET cycles were conducted, resulting in 76 196 pregnancies and 55 503 neonates. Single ET was performed in 81.6% of fresh transfers and 85.1% of frozen–thawed cycles, respectively, resulting in over 97% singleton pregnancies/livebirths rates.

**Conclusion:**

Despite the COVID‐19 pandemic during 2020, the overall number of ART cycles and neonates born demonstrated only a slight decrease in 2020 compared with 2019.

## Graphical abstract

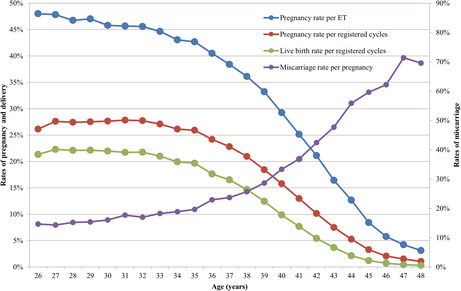

## 1 INTRODUCTION

Japanese women remain among the most significant users of assisted reproductive technology (ART) globally,^1^ and in 2019,^2^ the number of treatment cycles (458 101 treatment cycles) and neonates (60598) resulting from ART increased from that reported in 2018.^3^ Despite Japan having the highest utilization rate of ART, Japan's total fertility rate has progressively decreased over the past four decades.^4^ In 2019, the total fertility rate in Japan was 1.36, reaching a record low as reported by the Ministry of Health, Labour and Welfare, compared with 1.44 reported in 2016^1^ and 2.4 reported globally by the World Bank.^5^ The total fertility rate in 2020 remained low at 1.33 (fixed), and in 2021, it decreased further to 1.30 (based on monthly annual report calculations).^6^ Along with the decreasing trend of the total fertility rate and the number of neonates born in Japan, the proportion of neonates born from ART has been increasing.

Since 1986, the ART registry system of the Japan Society of Obstetrics and Gynecology (JSOG), and the online registration system implemented in 2007, have been collecting data on national trends of ART use and outcomes to understand the current effectiveness of ART, ensure ART safety, and inform decision‐making related to ART in Japan.^2^ The present report aims to summarize the data on characteristics and outcomes of registered ART cycles during 2020 and to compare the present results with results from previous years.

## 2 MATERIALS AND METHODS

### 2.1 Data source and data collection

The JSOG registry requested ART facilities across Japan to register data on the demographic and background characteristics of patients, clinical information including infertility diagnosis, pregnancy history, and delivery outcomes, and ART‐cycle specific data since 2007.^6^ The present descriptive analysis investigated registered cycle characteristics and treatment outcomes using data from the Japanese ART registry in 2020, with a cutoff date of 30 November 2021.

### 2.2 Variables of interest

Data for the following variables by fertilization method (in vitro fertilization [IVF], intracytoplasmic sperm injection [ICSI], and frozen–thawed embryo transfer [FET]) were collected, analyzed, and compared with data from previous years: Numbers of registered cycles, oocyte retrievals, embryo transfer (ET) cycles, freeze‐all‐embryo/oocyte cycle, pregnancies, and neonates. Characteristics of registered cycles and pregnancy outcomes were described for fresh, FET, and embryo transfers using frozen–thawed oocyte cycles. Fresh cycle data were stratified by fertilization method (i.e., IVF, ICSI, and gamete intrafallopian transfer (GIFT)).

### 2.3 Outcomes

The treatment outcomes analyzed and compared were as follows: pregnancy, defined as confirmation of a gestational sac in utero; miscarriage, defined as spontaneous or unplanned loss of a fetus from the uterus before 22 weeks of gestation; live birth, defined as delivery of at least one live neonate after 22 weeks of gestation; and multiple pregnancy rates. The pregnancy outcomes analyzed and compared were as follows: ectopic pregnancy, heterotopic pregnancy, artificially induced abortion, stillbirth, and fetal reduction. The following outcomes were also analyzed by maternal age: pregnancy, live birth, miscarriage, and multiple pregnancy rates. Treatment outcomes for FET cycles using frozen–thawed oocytes were also analyzed.

### 2.4 Statistical analysis

All analyses were conducted using the STATA MP statistical package, version 17.0 (Stata, College Station). Since the study focuses on descriptive analysis, statistical testing was not conducted.

## 3 RESULTS

The number of ART facilities participating in the registry in 2020 was 621 out of a total of 622 registered ART facilities in Japan. Among the 621 facilities participating in the registration, 20 did not implement any ART treatment.

Table 1 summarizes the main trends in the numbers of registered cycles, egg retrievals, pregnancy, and neonate births categorized by IVF, ICSI, and FET cycles in Japan (2007–2020). In 2020, 449 900 cycles were registered, and 60 381 births were recorded (1.79% and 0.36% decrease compared with the previous year). Notably, the number of registered IVF and ICSI cycles decreased by 5.9% and 2.0% from the previous year, respectively. The number of freeze‐all IVF and ICSI cycles increased in 2020, and the number of neonates born was 2282 for IVF‐ET cycles and 2596 for ICSI cycles, both of which were decreased from the previous year (23.3% and 24.3% decrease, respectively). Conversely, the number of FET cycles has continuously increased since 2007; however, the increase from 2019 was small at 0.04%, much lower than the increase of 5.8% from 2018 to 2019. In 2020, the number of FET cycles was 215 285, resulting in 76 196 pregnancies and 55 503 neonates (1.7% and 2.4% increase, respectively).

**TABLE 1:** Trends in numbers of registered cycles, oocyte retrieval, pregnancy and neonates based on IVF, ICSI and frozen–thawed embryo transfer cycles in Japan, 2007–2020

	IVF^a^						ICSI^b^						FET cycle^c^			
Year	No. of registered cycles	No. of egg retrievals	No. of freeze‐all cycles	No. of ET cycles	No. of cycles with pregnancy	No. of neonates	No. of registered cycles	No. of egg retrievals	No. of freeze‐all cycles	No. of ET cycles	No. of cycles with pregnancy	No. of neonates	No. of registered cycles	No. of ET cycles	No. of cycles with pregnancy	No. of neonates
2007	53 873	52 165	7626	28 228	7416	5144	61 813	60 294	11 541	34 032	7784	5194	45 478	43 589	13 965	9257
2008	59 148	57 217	10 139	29 124	6897	4664	71 350	69 864	15 390	34 425	7017	4615	60 115	57 846	18 597	12 425
2009	63 083	60 754	11 800	28 559	6891	5046	76 790	75 340	19 046	35 167	7330	5180	73 927	71 367	23 216	16 454
2010	67 714	64 966	13 843	27 905	6556	4657	90 677	88 822	24 379	37 172	7699	5277	83 770	81 300	27 382	19 011
2011	71 422	68 651	16 202	27 284	6341	4546	102 473	100 518	30 773	38 098	7601	5415	95 764	92 782	31 721	22 465
2012	82 108	79 434	20 627	29 693	6703	4740	125 229	122 962	41 943	40 829	7947	5498	119 089	116 176	39 106	27 715
2013	89 950	87 104	25 085	30 164	6817	4776	134 871	134 871	49 316	41 150	8027	5630	141 335	138 249	45 392	32 148
2014	92 269	89 397	27 624	30 414	6970	5025	144 247	141 888	55 851	41 437	8122	5702	157 229	153 977	51 458	36 595
2015	93 614	91 079	30 498	28 858	6478	4629	155 797	153 639	63 660	41 396	8169	5761	174 740	171 495	56 888	40 611
2016	94 566	92 185	34 188	26 182	5903	4266	161 262	159 214	70 387	38 315	7324	5166	191 962	188 338	62 749	44 678
2017	91 516	89 447	36 441	22 423	5182	3731	157 709	155 758	74 200	33 297	6757	4826	198 985	195 559	67 255	48 060
2018	92 552	90 376	38 882	20 894	4755	3402	158 859	157 026	79 496	29 569	5886	4194	203 482	200 050	69 395	49 383
2019	88 074	86 334	40 561	17 345	4002	2974	154 824	153 014	83 129	24 490	4789	3433	215 203	211 758	74 911	54 188
2020	82 883	81 286	42 530	13 362	3094	2282	151 732	150 082	87 697	19 061	3626	2596	215 285	211 914	76 196	55 503

Abbreviations: ET, embryo transfer; FET, frozen–thawed embryo transfer; GIFT, gamete intrafallopian transfer; ICSI, intracytoplasmic sperm injection; IVF, in vitro fertilization.

^a^ Including GIFT and other.

^b^ Including split‐ICSI cycles.

^c^ Including cycles using frozen–thawed oocyte.

**FIGURE 1:**
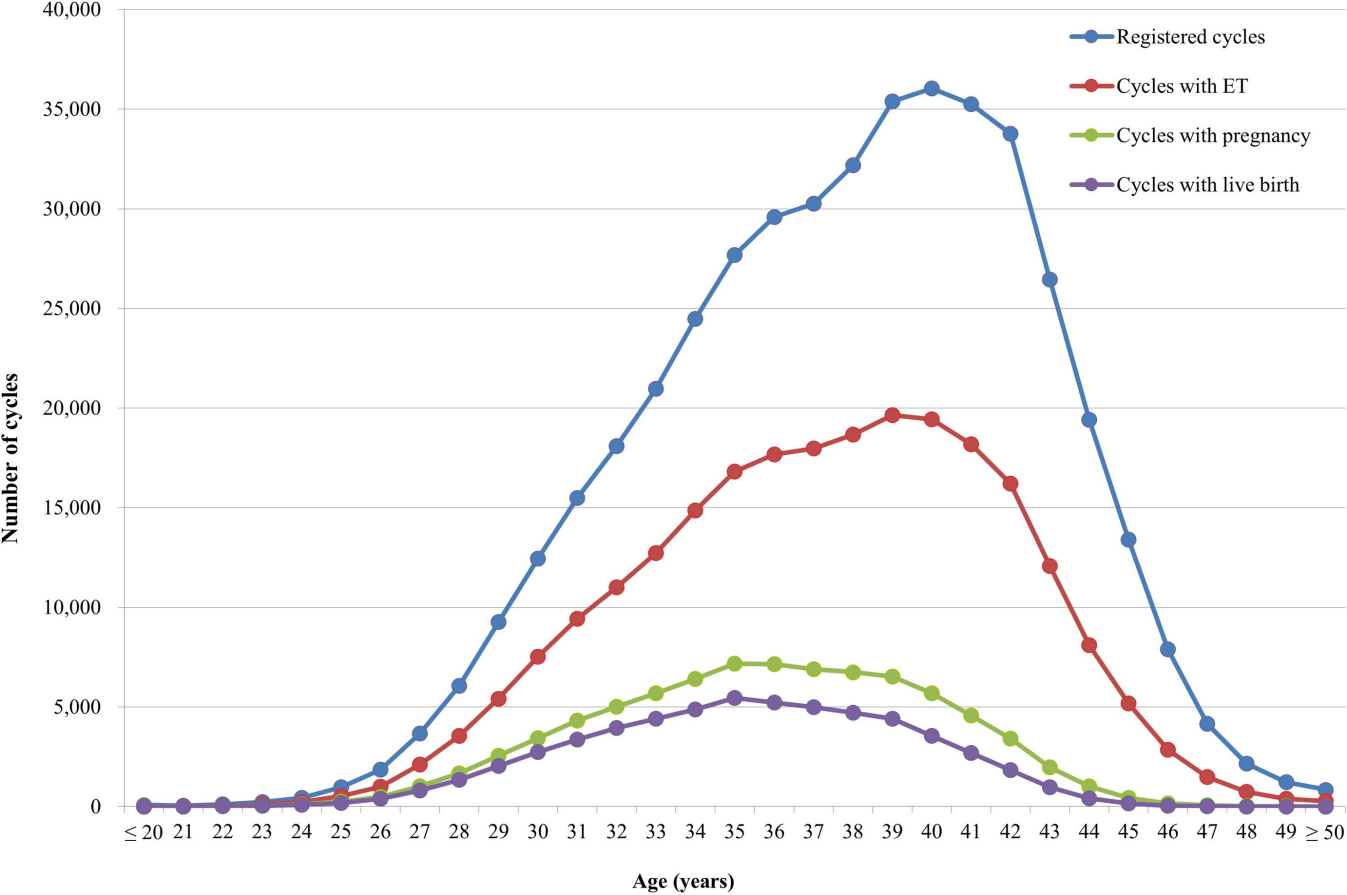
Distribution of maternal age from all registered cycles, cycles for ET, cycles leading to pregnancy and live births in 2020. Adapted from the Japan Society of Obstetrics and Gynecology ART Databook 2020 (https://www.jsog.or.jp/activity/art/2020data_202208.pdf). ET, embryo transfer

Figure 1 shows the age distributions for all registered cycles and different subgroups of cycles for ET, pregnancy, and live births in 2020. The mean patient age for registered cycles was 37.8 years (standard deviation [SD] ± 4.8); the mean age for pregnancy and live birth cycles was 35.8 years (SD ± 4.2) and 35.3 years (SD ± 4.1), respectively. Notably, 40.1% of ART cycles registered in 2020 were undertaken for women aged 40 years or over.

### 3.1 Treatment and pregnancy outcomes

The detailed characteristics and treatment outcomes of registered fresh cycles are shown in Table 2. In 2020, 77 543 IVF cycles, 28 986 split‐ICSI cycles, 120 840 ICSI cycles using ejaculated spermatozoa, 1906 ICSI cycles using testicular sperm extraction (TESE), 8 GIFT cycles, 894 cycles for oocyte freezing, and 4438 other cycles were registered. In total, 231 368 cycles resulted in oocyte retrieval, of which 130 227 (56.3%) were freeze‐all cycles. For IVF, the pregnancy rate per ET cycle was 23.1%, and for ICSI using ejaculated spermatozoa was 18.0%. The total single ET rate was 81.6%, and the pregnancy rate following a single ET cycle was 21.1%. Live birth rates per ET were 16.7% for IVF, 18.3% for split‐ICSI, 12.4% for ICSI using ejaculated spermatozoa, 9.7% for ICSI with TESE, and 12.5% for GIFT. There were 6404 singleton pregnancies and 4633 singleton live births. The rate of singleton pregnancies was 97.6%, and the rate of singleton live births was 97.3%. In total, 894 cycles for oocyte freezing were registered, and 882 oocyte retrievals were conducted. Of these, 757 cycles led to successfully frozen oocytes.

**TABLE 2:** Characteristics and treatment outcomes of registered fresh cycles in assisted reproductive technology in Japan, 2020

Variables	IVF	Split‐ICSI	ICSI		GIFT	Frozen oocyte	Other^a^	Total
			Ejaculated sperm	TESE				
No. of registered cycles	77 543	28 986	120 840	1906	8	894	4438	234 615
No. of egg retrievals (0 or more)	76 068	28 734	119 445	1903	8	882	4328	231 368
No. of fresh ET cycles (1 or more)	13 093	2952	15 821	288	8	0	261	32 423
No. of freeze‐all cycles	39 917	22 754	63 792	1151	0	757	1856	130 227
No. of cycles with pregnancy	3028	734	2849	43	1	0	65	6720
Pregnancy rate per ET	23.1%	24.9%	18.0%	14.9%	12.5%		24.9%	20.7%
Pregnancy rate per egg retrieval	4.0%	2.6%	2.4%	2.3%	12.5%		1.5%	2.9%
Pregnancy rate per egg retrieval excluding freeze‐all cycles	8.4%	12.3%	5.1%	5.7%	12.5%		2.6%	6.6%
SET cycles	11 038	2512	12 499	190	1		223	26 463
Pregnancy following SET cycles	2560	629	2304	30	0		60	5583
Rate of SET cycles	84.3%	85.1%	79.0%	66.0%	12.5%		85.4%	81.6%
Pregnancy rate following SET cycles	23.2%	25.0%	18.4%	15.8%	0.0%		26.9%	21.1%
Miscarriages	741	156	791	15	0		10	1713
Miscarriage rate per pregnancy	24.5%	21.3%	27.8%	34.9%	0.0%		15.4%	25.5%
Singleton pregnancies^b^	2910	701	2690	38	1		64	6404
Multiple pregnancies^b^	72	14	85	1	0		0	172
Twin pregnancies	72	14	83	1	0		0	170
Triplet pregnancies	0	0	2	0	0		0	2
Quadruplet pregnancies	0	0	0	0	0		0	0
Multiple pregnancy rate	2.4%	2.0%	3.1%	2.6%	0.0%		0.0%	2.6%
Live births	2181	540	1957	28	1		53	4760
Live birth rate per ET	16.7%	18.3%	12.4%	9.7%	12.5%		20.3%	14.7%
Total no. of neonates	2228	556	2012	28	1		53	4878
Singleton live births	2128	524	1899	28	1		53	4633
Twin live births	50	16	55	0	0		0	121
Triplet live births	0	0	1	0	0		0	1
Quadruplet live births	0	0	0	0	0		0	0
Ectopic pregnancies	20	16	33	0	0		1	70
Heterotopic pregnancies	1	1	0	0	0		0	2
Artificial abortions	14	5	14	0	0		0	33
Still births	10	2	7	0	0		0	19
Fetal reductions	0	0	0	0	0		0	0
Cycles with unknown pregnancy outcomes	44	8	27	0	0		1	80

Abbreviations: ET, embryo transfer; GIFT, gamete intrafallopian transfer; ICSI, intracytoplasmic sperm injection; IVF, in vitro fertilization; SET, single embryo transfer; TESE, testicular sperm extraction; ZIFT, zygote intrafallopian transfer.

^a^ Others include ZIFT.

^b^ Singleton, twin, triplet, and quadruplet pregnancies were defined on the basis of the number of gestational sacs in utero.

Table 3 summarizes the characteristics and treatment outcomes of FET cycles. In 2020, 214 990 cycles were registered. Of these, FET was registered in 214 153 cycles, of which 211 042 FETs were actually conducted. With a pregnancy rate of 36.0%, FET cycles resulted in 75 981 pregnancies. FET cycles resulted in 18 852 miscarriages. The miscarriage rate per pregnancy was 24.8%, and the live birth rate per FET was 25.5%. The single ET rate was 85.1%, resulting in a pregnancy rate of 37.1%. The respective singleton pregnancy and live birth rates were 97.0% respectively.

**TABLE 3:** Characteristics and treatment outcomes of frozen cycles in assisted reproductive technology in Japan, 2020

Variables	FET	Other^a^	Total
No. of registered cycles	214 153	837	214 990
No. of FET	211 042	717	211 759
No. of cycles of pregnancy	75 981	170	76 151
Pregnancy rate per FET	36.0%	23.7%	36.0%
SET cycles	179 609	567	180 176
Pregnancy following SET cycles	66 552	137	66 689
Rate of SET cycles	85.1%	79.1%	85.1%
Pregnancy rate following SET cycles	37.1%	24.2%	37.0%
Miscarriages	18 852	49	18 901
Miscarriage rate per pregnancy	24.8%	28.8%	24.8%
Singleton pregnancies^b^	72 457	164	72 621
Multiple pregnancies^b^	2253	6	2259
Twin pregnancies	2195	6	2201
Triplet pregnancies	58	0	58
Quadruplet pregnancies	0	0	0
Multiple pregnancy rate	3.0%	3.5%	3.0%
Live births	53 891	111	54 002
Live birth rate per FET	25.5%	15.5%	25.5%
Total no. of neonates	55 349	114	55 463
Singleton live births	52 286	108	52 394
Twin live births	1500	3	1503
Triplet live births	21	0	21
Quadruplet live births	0	0	0
Ectopic pregnancies	419	0	419
Heterotopic pregnancies	8	0	8
Artificial abortions	352	3	355
Still births	200	0	200
Fetal reductions	13	0	13
Cycles with unknown pregnancy outcomes	1659	7	1666

Abbreviations: FET, frozen–thawed embryo transfer; SET, single embryo transfer.

^a^ Including cycles using frozen–thawed oocytes.

^b^ Singleton, twin, triplet, and quadruplet pregnancies were defined on the basis of the number of gestational sacs in utero.

### 3.2 Outcomes by maternal age

**FIGURE 2:**
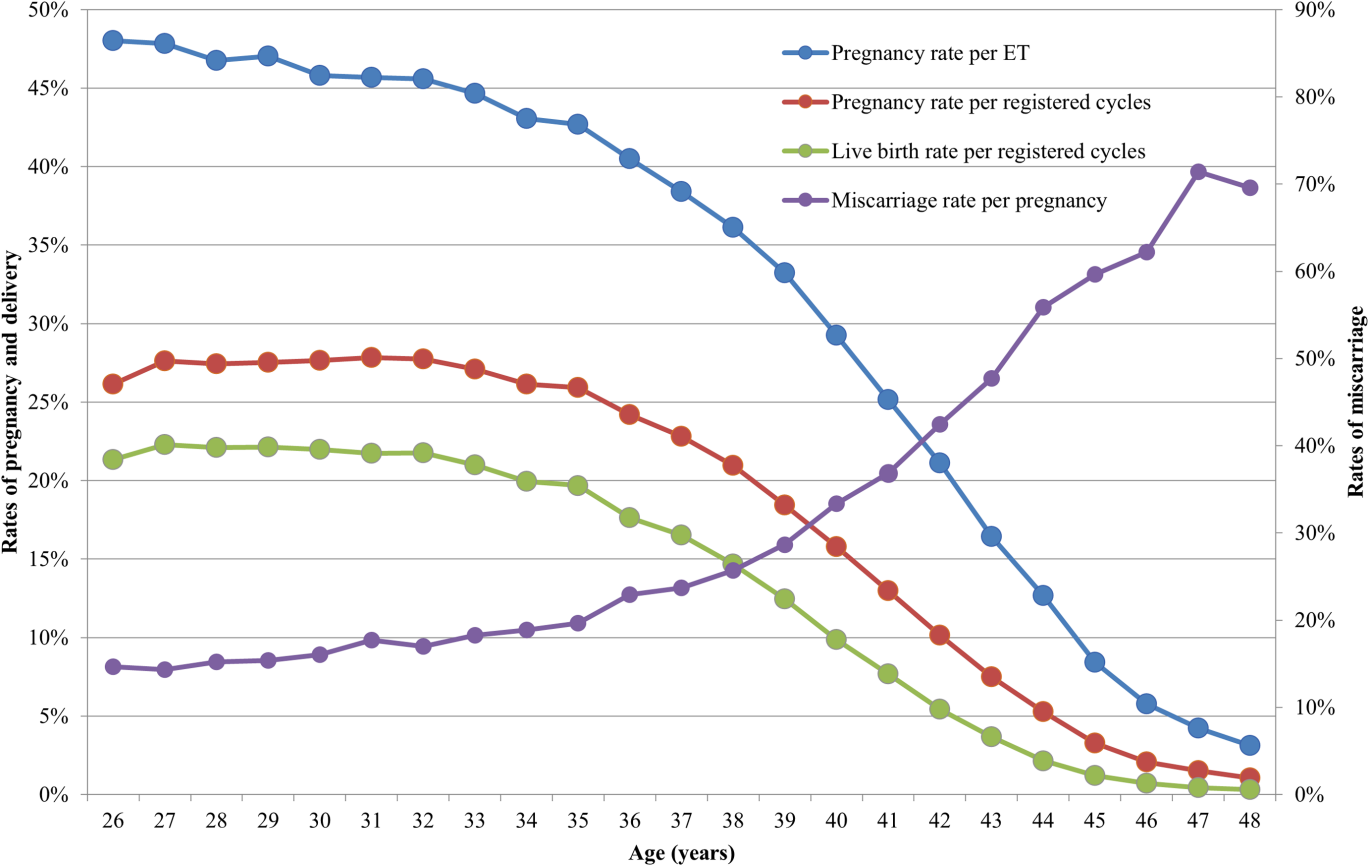
Pregnancy, live birth, and miscarriage rates according to patient age in all registered cycles in 2019. Adapted from the Japan Society of Obstetrics and Gynecology ART Databook 2020 (https://www.jsog.or.jp/activity/art/2020data_202208.pdf). ET, embryo transfer

Table 4 shows the treatment outcomes of registered cycles by maternal age in 2020. Figure 2 shows the pregnancy, live birth, and miscarriage rates by maternal age in all registered cycles in 2020.

**TABLE 4:** Treatment outcomes of registered cycles based on patient age in Japan, 2020

Age (years)	No. of registered cycles	No. of ET cycles	No. of cycles with pregnancy	Multiple pregnancies	Miscarriage	Cycles with live birth	Pregnancy rate/registered ET	Pregnancy rate/registered cycles	Live birth rate/registered cycles	Miscarriage rate /pregnancy	Multiple pregnancy rate^a^
≤20	80	4	1	0	0	1	20.0%	1.3%	1.3%	0.0%	0.0%
21	42	14	6	1	1	5	42.9%	14.3%	11.9%	16.7%	16.7%
22	123	47	22	1	0	22	46.8%	17.9%	17.9%	0.0%	4.6%
23	227	125	55	3	9	43	44.0%	24.2%	18.9%	16.4%	5.6%
24	447	241	114	1	14	92	47.3%	25.5%	20.6%	12.3%	0.90%
25	978	542	248	6	44	195	45.8%	25.4%	19.9%	17.7%	2.5%
26	1852	1008	484	17	71	395	48.0%	26.1%	21.3%	14.7%	3.60%
27	3661	2114	1011	19	145	816	47.8%	27.6%	22.3%	14.3%	1.9%
28	6056	3554	1662	42	253	1338	46.8%	27.4%	22.1%	15.2%	2.6%
29	9269	5421	2550	75	392	2051	47.0%	27.5%	22.1%	15.4%	3.0%
30	12 451	7521	3444	100	553	2736	45.8%	27.7%	22.0%	16.1%	3.0%
31	15 486	9432	4309	130	764	3365	45.7%	27.8%	21.7%	17.7%	3.1%
32	18 083	11 008	5017	131	851	3937	45.6%	27.7%	21.8%	17.0%	2.7%
33	20 976	12 728	5684	148	1039	4405	44.7%	27.1%	21.0%	18.3%	2.7%
34	24 485	14 866	6403	187	1207	4882	43.1%	26.2%	19.9%	18.9%	3.0%
35	27 685	16 805	7176	224	1409	5449	42.7%	25.9%	19.7%	19.6%	3.2%
36	29 582	17 678	7158	228	1641	5213	40.5%	24.2%	17.6%	22.9%	3.2%
37	30 261	17 981	6905	227	1635	5002	38.4%	22.8%	16.5%	23.7%	3.3%
38	32 175	18 672	6745	188	1734	4719	36.1%	21.0%	14.7%	25.7%	2.8%
39	35 398	19 638	6525	208	1870	4406	33.2%	18.4%	12.4%	28.7%	3.2%
40	36 049	19 446	5686	177	1896	3559	29.2%	15.8%	9.9%	33.3%	3.2%
41	35 237	18 176	4574	154	1684	2702	25.2%	13.0%	7.7%	36.8%	3.4%
42	33 771	16 212	3422	70	1452	1830	21.1%	10.1%	5.4%	42.4%	2.1%
43	26 438	12 071	1982	59	946	970	16.4%	7.5%	3.7%	47.7%	3.0%
44	19 423	8112	1027	27	574	417	12.7%	5.3%	2.1%	55.9%	2.7%
45	13 387	5184	436	5	260	162	8.4%	3.3%	1.2%	59.6%	1.2%
46	7898	2848	164	2	102	56	5.8%	2.1%	0.70%	62.2%	1.2%
47	4150	1488	63	1	45	18	4.2%	1.5%	0.40%	71.4%	1.6%
48	2157	739	23	0	16	7	3.1%	1.1%	0.30%	69.6%	0.0%
49	1226	384	12	0	8	4	3.1%	1.0%	0.30%	66.7%	0.0%
≥50	847	278	8	1	4	3	2.9%	0.90%	0.40%	50.0%	12.5%
Total	449 900	244 337	82 916	2432	20 619	58 800	33.9%	18.4%	13.1%	24.9%	3.0%

Abbreviation: ET, embryo transfer.

^a^ Multiple pregnancies were defined on the basis of the number of gestational sacs in utero.

The pregnancy rate per ET exceeded 40% for maternal ages between 21 and 36 years. Gradual decreases in pregnancy rates per ET were observed with increasing maternal age, with rates falling below 30% for women aged between 40 and 42 years of age, rates below 20% among women aged >43 years of age, reaching rates below 10% for women >45 years of age. The miscarriage rates were below 20% for all women <35 years of age and increased gradually with increasing maternal age. Women in their early forties had miscarriage rates of between 33.3% and 47.7%, while women in their mid‐forties or older had miscarriage rates over 59.6%. Live birth rates per registered cycle were between 30% and 35% for women between 24 and 33 years of age, which declined sharply to below 20% at 39 years of age and below 10% among women >41 years of age.

### 3.3 Treatment outcomes for FET cycles using frozen–thawed oocytes

Table 5 summarizes the treatment outcomes of embryo transfers using frozen–thawed oocytes in Japan in 2020. A total of 295 cycles using frozen–thawed oocytes were registered in Japan in 2020, and 155 FETs were actually implemented. Forty‐five pregnancies were achieved, with a pregnancy rate per FET of 29.0% and a live birth rate of 24.5%. The miscarriage rate per pregnancy was 11.1%.

**TABLE 5:** Treatment outcomes of embryo transfers using frozen–thawed oocyte in assisted reproductive technology in Japan, 2020

Variables	Embryo transfers using frozen–thawed oocytes
No. of registered cycles	295
No. of ET	155
No. of cycles with pregnancy	45
Pregnancy rate per ET	29.0%
SET cycles	99
Pregnancy following SET cycles	25
Rate of SET cycles	63.9%
Pregnancy rate following SET cycles	25.3%
Miscarriages	5
Miscarriage rate per pregnancy	11.1%
Singleton pregnancies^a^	44
Multiple pregnancies^a^	1
Twin pregnancies	1
Triplet pregnancies	0
Quadruplet pregnancies	0
Multiple pregnancy rate	2.2%
Live births	38
Live birth rate per ET	24.5%
Total number of neonates	40
Singleton live births	36
Twin live births	2
Triplet live births	0
Quadruplet live births	0
Ectopic pregnancies	1
Intrauterine pregnancies coexisting with ectopic pregnancy	0
Artificial abortions	0
Still births	0
Fetal reductions	0
Cycles with unknown pregnancy outcomes	1

Abbreviations: ET, embryo transfer; SET, single embryo transfer.

^a^ Singleton, twin, triplet, and quadruplet pregnancies were defined on the basis of the number of gestational sacs in utero.

## 4 DISCUSSION

In this report, we describe the characteristics and outcomes of ART cycles registered in the Japanese ART registry system during 2020 and compare the present results with those from 2019^2^ and previous years.^3^ In 2020, the JSOG registered a total of 449 900 ART cycles in Japan, which resulted in the birth of 60 381 neonates. Of note, these numbers are slightly lower than the 458 101 cycles and 60 598 neonates reported in 2019 (1.79% and 0.36% decrease, respectively).^2^ Compared with the 2019 report, the number of fresh cycles (including IVF and ICSI) decreased in 2020, in line with the downward trend observed in the number of fresh IVF and ICSI cycles from 2018^3^ to 2019.^2^ In contrast, the number of freeze‐all cycles of IVF and ICSI continued to increase in line with the trend observed over the past 6 years.^2^, ^3^, ^7^, ^8^, ^9^, ^10^ As a result, the number of live births resulting from IVF‐ET and ICSI cycles decreased even further (2282 for IVF‐ET cycles and 2596 for ICSI cycles) than that reported in the previous year (2974 and 3433, respectively)^5^ and 2018 (3402 and 4194, respectively).^3^

FET cycles continued to increase in contrast with the previous years, but the increase was slight (+0.04%) compared with 2019 (+5.8%). FET cycles contributed to 215 285 cycles (from 215 203 in 2019), 76 196 pregnancies, and 55 503 neonate births. The total single ET rates were similar for fresh (81.6%) and frozen (85.1%) cycles and comparable with those of the previous year (82.6% and 85.1%).^2^ Live birth rates per ET for fresh and frozen cycles remained largely unchanged from 2019. The rates of singleton pregnancies and singleton live births for fresh (97.6% and 97.3%) and frozen cycles (97.4% and 97.0%, respectively) were similar and aligned with those reported in 2019 (97.3% and 97.3% for fresh and 97.1% and 97.3% for frozen cycles).^2^

Regarding the distribution of outcomes per maternal age, the mean age of patients undergoing ART procedures in Japan in 2020 was 37.8 years (SD ± 4.8). In keeping with the trends reported in 2019 (40.7%) and 2018 (41.8%), 40.1% of ART cycles registered in 2020 were undertaken by women ≥40 years of age. Consistent with the previous report, the pregnancy rates achieved with ART were over 40% among women between 21 and 36 years of age, the pregnancy rate declined progressively with increasing age in women >36 years, and the miscarriage rates increased progressively with increasing age in women >36 years. Live birth rates per registered cycle were between 30% and 35% for women between 24 and 33 years of age, and these declined sharply to below 20% at >39 years of age.

This yearly analysis is crucial for understanding trends and patterns in ART, which are highly relevant given the continuously declining total fertility rate, increasing aging population, and decreasing population growth in Japan.^11^, ^12^, ^13^, ^14^ Additionally, yearly data can show changes in ART use and outcome patterns resulting from policy and insurance coverage changes. For instance, an expansion of insurance coverage for ART, including IVF and ICSI, as well as embryo freezing and ET, is a recent measure adopted by the Japanese government to aid couples with infertility in Japan as a form of support and incentive to undergo ART.^15^ As this measure has been implemented relatively recently (April 2022),^15^ the long‐term effects that this measure may have on the usage of ART services and its impact on total fertility rates in Japan are still unknown. Thus, after implementing the Japanese government health coverage for ART, it is essential to evaluate the results with those of previous years and discuss the impact on future ART treatment patterns and outcomes. In contrast with the previous countermeasures, i.e., assistance for childcare and additional support for a work‐life balance, subsidies, and incentives for ART treatments (no longer available since coverage was provided in April 2022), it is expected that insurance coverage of ART may provide a benefit for couples experiencing fertility issues by improving accessibility of ART. A recent Japan Society for Reproductive Medicine (JSRM) guideline update aims to standardize the infertility treatments to be covered, which are those recommended in the treatment guidelines.^16^

Of note, on 11 March 2020, the World Health Organization declared the novel coronavirus (COVID‐19) outbreak a global pandemic.^17^ On 1 April 2020, the JSRM issued a statement recommending that ART treatments be postponed because of concerns about the COVID‐19 pandemic and the state of emergency announced by the Japanese government.^18^ On 18 May 2020, the state of emergency was lifted in 39 Japanese prefectures, and the JSRM issued another statement advising clinicians to resume ART treatments while taking appropriate measures to prevent COVID‐19.^19^ During 2020, the situation varied widely with different levels of restrictions applied in different countries, but the situation in Japan was unique as there was no mandated nationwide lockdown, rather a voluntary stay‐at‐home measure. The Japanese government continued to urge the general public to practice social distancing and avoid contact unless necessary to address essential or urgent matters.^20^, ^21^ Because of such restrictions, ART procedures, which may have been deemed non‐essential or non‐urgent, were expected to decrease in 2020.

The COVID‐19 pandemic affected ART treatment cycles worldwide by causing the cessation of new treatments, delays and postponements of treatment, and changes or interruptions of the stimulation protocols for couples undergoing ART.^22^, ^23^, ^24^, ^25^, ^26^, ^27^ In Japan, literature on the effects of the COVID‐19 pandemic on ART facilities, services, outcomes, and patients is limited and detailed analyses have yet to be conducted. However, the COVID‐19 pandemic was considered to have little effect on decreasing the total number of ART cycles implemented during 2020 compared with other European countries. It is likely that the number of ART cycles temporally decreased once the JSRM statement was emitted but caught up rapidly by the end of the year.

In 2021 Tsutsumi et al. reported that at Sanno Hospital, the number of ETs was temporarily decreased, but the number of oocyte retrievals increased. However, many patients wished to continue ART treatments while taking appropriate measures to prevent COVID‐19 infection and any potential sequelae to the mother and neonate.^28^ Similar concerns and desires were voiced by patients in other countries who also experienced cancellations or delays in ART cycles.^25^, ^26^ After the first statement issued by the JSRM to patients, a web questionnaire survey conducted in August 2020 by a non‐profit organization in support of patients with infertility issues revealed that many patients were concerned about COVID‐19. Many wanted to continue ART treatments, especially those aged >40 years, and a large number of patients asserted that infertility treatments are not “non‐essential and non‐urgent” matters, which posed an important ethical dilemma at the time.^29^ Although it has been discussed that such COVID‐19‐related delays and cancellations of ART cycles should not have affected treatment outcomes, it is possible that such measures may have contributed to the lower number of registered cycles, pregnancy rates, and live births compared with 2019.

This study has some strengths and limitations. The strengths include high reporting compliance as reporting is mandatory for designated ART facilities nationwide, and designated ART facilities use standardized definitions for cycle‐specific information, thus reducing reporting bias. The main limitation was the missing background information, for which collection was not standardized. For instance, significant proportions of data on body mass index, number of previous pregnancies and parity, husband's age, and patient's height and weight were still unavailable. Therefore, establishing systems to decrease the missingness rate for these variables would be necessary to improve the accuracy of analyses of these data. Furthermore, other background information, such as the presence of relevant patient conditions (e.g., polycystic ovarian syndrome and poor ovarian reserve) and relatively new treatments, such as preimplantation genetic testing and progestin‐primed ovarian stimulation, should also be collected, as these may have a significant impact on ART outcomes. These data will be included in the registries from January 2022. Because the data collection for the registry was carried out per cycle, we could not distinguish whether a person received multiple treatment cycles.

To conclude, the 2020 analysis of the ART registry showed that despite the COVID‐19 pandemic during 2020, the overall number of ART cycles and neonates born demonstrated a slight decrease in 2020 compared with 2019, while the number of FETs maintained an increasing trend in 2020 (a small increase of 0.04% from 2019). The rate of single ETs, both fresh and frozen, also increased in 2020, but the increase was slightly higher for FET cycles. For both fresh and FET cycles, the rates of singleton pregnancies and live births were over 97%, similar to those in 2019. The ART use and outcomes trends in Japan in 2020 were similar to those of the previous year. It will be interesting to compare these results with future results after the recent implementation of the Japanese government health coverage for ART.

## CONFLICT OF INTEREST

The authors have no conflict of interest to disclose in relation with the present work.

## ETHICAL APPROVAL

Not applicable.

## HUMAN RIGHTS STATEMENTS AND INFORMED CONSENT

All procedures were performed in accordance with the ethical standards of the relevant committees on human experimentation (institutional and national) and the Helsinki Declaration of 1964 and its later amendments.

## ANIMAL RIGHTS

This report does not contain any studies performed by any authors that included animals.

## CLINICAL TRIAL REGISTRY

Not applicable.

## TRIAL REGISTRATION

NONE.

## References

[1] Chambers GM , Dyer S , Zegers‐Hochschild F , de Mouzon J , Ishihara O , Banker M , et al. International committee for monitoring assisted reproductive technologies world report: assisted reproductive technology, 2014. Hum Reprod. 2021;36(11):2921–34. 34601605 10.1093/humrep/deab198

[2] Katagiri Y , Jwa SC , Kuwahara A , Iwasa T , Ono M , Kato K , et al. Assisted reproductive technology in Japan: a summary report for 2019 by the ethics Committee of the Japan Society of obstetrics and gynecology. Reprod Med Biol. 2021;21(1):e12434. 35386377 10.1002/rmb2.12434 PMC8967301

[3] Ishihara O , Jwa SC , Kuwahara A , Katagiri Y , Kuwabara Y , Hamatani T , et al. Assisted reproductive technology in Japan: a summary report for 2018 by the ethics Committee of the Japan Society of obstetrics and gynecology. Reprod Med Biol. 2020;20(1):3–12. 33488278 10.1002/rmb2.12358 PMC7812461

[4] Ministry of Health, Labour and Welfare of Japan . Handbook of health and welfare statistics 2021 contents. [Internet] Part 1 Population and households: Table 1‐20. Total fertility rates by year. Tokyo: Ministry of Health, Labour and Welfare of Japan;2022. Available from: https://www.mhlw.go.jp/english/database/db‐hh/1‐2.html

[5] The World Bank . Fertility rate, total (births per woman), 2021. [Internet] [Cited 2022 Oct 10] Available from: https://data.worldbank.org/indicator/SP.DYN.TFRT.IN

[6] Ministry of Health, Labour and Welfare of Japan . Demographic statistics monthly‐annual report calculation. Tokyo: Ministry of Health, Labour and Welfare of Japan [Internet] [Cited 2022 Oct 10] Available from: https://www.mhlw.go.jp/toukei/saikin/hw/jinkou/geppo/nengai21/dl/gaikyour3.pdf

[7] Irahara M , Kuwahara A , Iwasa T , Ishikawa T , Ishihara O , Kugu K , et al. Assisted reproductive technology in Japan: a summary report of 1992‐2014 by the ethics committee, Japan Society of Obstetrics and Gynecology. Reprod Med Biol. 2017;16(2):126–32. 29259459 10.1002/rmb2.12014 PMC5661813

[8] Saito H , Jwa SC , Kuwahara A , Saito K , Ishikawa T , Ishihara O , et al. Assisted reproductive technology in Japan: a summary report for 2015 by the ethics Committee of the Japan Society of Obstetrics and Gynecology. Reprod Med Biol. 2017;17(1):20–8. 29371817 10.1002/rmb2.12074 PMC5768979

[9] Ishihara O , Jwa SC , Kuwahara A , Ishikawa T , Kugu K , Sawa R , et al. Assisted reproductive technology in Japan: a summary report for 2016 by the ethics Committee of the Japan Society of obstetrics and gynecology. Reprod Med Biol. 2018;18(1):7–16. 30655717 10.1002/rmb2.12258 PMC6332769

[10] Ishihara O , Jwa SC , Kuwahara A , Katagiri Y , Kuwabara Y , Hamatani T , et al. Assisted reproductive technology in Japan: a summary report for 2017 by the ethics Committee of the Japan Society of obstetrics and gynecology. Reprod Med Biol. 2019;19(1):3–12. 31956280 10.1002/rmb2.12307 PMC6955594

[11] National Institute of Population and Social Security Research . Population projections for Japan: Tokyo: 2016 to 2065. 2017. [Internet] [Cited 2022 Oct 10] Available from: http://www.ipss.go.jp/index‐e.asp

[12] Matsuda S . In: Matsuda S , editor. Low fertility in Japan, South Korea, and Singapore: population policies and their effectiveness. 1st ed. London: Springer Nature; 2020. p. 1–14.

[13] Ghaznavi C , Sakamoto H , Yamasaki L , Nomura S , Yoneoka D , Shibuya K , et al. Salaries, degrees, and babies: trends in fertility by income and education among Japanese men and women born 1943‐1975‐analysis of national surveys. PLoS One. 2022;17(4):e0266835. 35476638 10.1371/journal.pone.0266835 PMC9045600

[14] Schoppa LJ . The policy response to declining fertility rates in Japan: relying on logic and hope over evidence. Soc Sci Japan J. 2020;23(1):3–21.

[15] Ministry of Health, Labour and Welfare of Japan . Infertility treatment is covered by insurance from April 2022. Tokyo: Ministry of Health, Labour and Welfare of Japan [Internet] [Cited 2022 Oct 10] Available from: https://www.mhlw.go.jp/content/leaflet202212ver2.pdf

[16] Japan Society of Reproductive Medicine . Information on publication of guidelines for reproductive medicine. [Internet] [Cited 2022 Oct 10] Available from http://www.jsrm.or.jp/publications/pub_guidelines.html

[17] World Health Organization . Coronavirus disease (COVID‐19) pandemic. [Internet] WHO; 2022. [Cited 2022 Oct 10] Available from. https://www.who.int/emergencies/diseases/novel‐coronavirus‐2019

[18] Japanese Society for Reproductive Medicine . Statement from the Japanese Society for reproductive medicine on novel coronavirus disease (COVID‐19). Tokyo: [Internet] JSRM; 2020. Cited 2022 Oct 10] Available from:. http://www.jsrm.or.jp/announce/187.pdf

[19] Japanese Society for Reproductive Medicine . Notification from the Japanese Society for Reproductive Medicine for novel coronavirus infectious disease (COVID‐19). Tokyo: [Internet] JSRM; 2020. [Cited 2022 Oct 10] Available from:. http://www.jsrm.or.jp/announce/195.pdf

[20] Watanabe T , Yabu T . Japan's voluntary lockdown. PLoS One. 2021;16(6):e0252468. 34111163 10.1371/journal.pone.0252468 PMC8191912

[21] Office for COVID‐19 and Other Emerging Infectious Disease Control, Cabinet Secretariat, Government of Japan . Measures to be taken based on the basic response policy. [Internet] Cabinet Secretariat. 2020. [Cited 2022 Oct 10] Available from: https://corona.go.jp/en/

[22] Gemmell LC , Williams Z , Forman EJ . Considerations on the restriction of assisted reproductive technology (ART) due to COVID‐19. Semin Perinatol. 2020;44(7):151288. 33317710 10.1016/j.semperi.2020.151288 PMC7450235

[23] Anifandis G , Messini CI , Simopoulou M , Sveronis G , Garas A , Daponte A , et al. SARS‐CoV‐2 vs. human gametes, embryos and cryopreservation. Syst Biol Reprod Med. 2021;67(4):260–9. 34060390 10.1080/19396368.2021.1922537

[24] Martins da Silva SJ , Campo‐Engelstein L . Assisted reproductive technology, justice and autonomy in an era of COVID‐19. Reprod Biomed Online. 2021;42(2):287–90. 33279418 10.1016/j.rbmo.2020.11.004 PMC7667398

[25] Schirmer AD , Kawwass JF , Adashi EY . Fertility care amidst the COVID19 pandemic: the American experience. J Ovarian Res. 2021;14(1):34. 33602259 10.1186/s13048-021-00782-4 PMC7890540

[26] Jirge PR , Patwardhan S , Jirge SN , et al. Resuming assisted reproduction services during COVID‐19 pandemic: an initial Indian experience. J Hum Reprod Sci. 2020;13(4):323–32. 33627983 10.4103/jhrs.JHRS_211_20 PMC7879848

[27] Banker M , Arora P , Banker J , Shah A , Gupta R , Shah S . Impact of COVID‐19 pandemic on clinical and embryological outcomes of assisted reproductive techniques. J Hum Reprod Sci. 2022;15(2):150–6. 35928469 10.4103/jhrs.jhrs_57_22 PMC9345275

[28] Tsutsumi O . COVID‐19 pandemic and fertility treatment. Obstet Gynecol Pract. 2021;70(2):159–66. [In Japanese].

[29] Fertility Information Network (Fine) . 2022. [Cited 2022 Oct 10] Available from: https://j‐fine.jp/

